# Pseudothrombocytopenia due to Phagocytosis of Platelets by Polymorphonuclear Leukocytes

**DOI:** 10.1002/ajh.27562

**Published:** 2024-12-16

**Authors:** Iliana Stamatiou, Zoe Bezirgiannidou, Evangelia Charitaki, Ioannis Kotsianidis, Konstantinos Liapis

**Affiliations:** ^1^ Second University Department of Internal Medicine University Hospital of Alexandroupolis, Democritus University of Thrace Alexandroupolis Greece; ^2^ Department of Hematology University Hospital of Alexandroupolis, Democritus University of Thrace Alexandroupolis Greece; ^3^ Department of Nephrology University Hospital of Alexandroupolis, Democritus University of Thrace Alexandroupolis Greece

1

A 75‐year‐old woman presented to the emergency department with progressive abdominal pain, fever, and diarrhea after taking levofloxacin for a respiratory tract infection. On evaluation, she was in clinical shock, with blood pressure 88/58 mmHg and heart rate 122 beats per minute. A complete blood count provided by the Sysmex XN‐1000 analyzer showed leukocytosis (13.8 × 10^9^/L, 95% neutrophils) and thrombocytopenia (22 × 10^9^/L). She had acidosis, renal impairment, coagulopathy, and elevated C‐reactive protein level. Because of the thrombocytopenia, an examination of a peripheral‐blood smear was performed in the hematology laboratory, which showed vacuolated neutrophils that contained phagocytized platelets. Of 200 neutrophils examined, 161 (80%) contained between one and six platelets (Figure 1). These findings indicated spurious thrombocytopenia. The emergency department staff were notified by the laboratory that the patient's platelet count was normal. Subsequently, she underwent internal jugular‐vein catheterization for fluid resuscitation without oozing or hematoma. Pseudomembranous colitis was diagnosed on the basis of a positive *Clostridioides difficile* stool test. She was treated with metronidazole and vancomycin, but her course was complicated by renal failure necessitating hemodialysis. Eventually, she made a full recovery. During hospitalization, multiple routinely prepared films from EDTA‐anticoagulated blood consistently demonstrated platelet phagocytosis but with the resolution of the colitis, the phenomenon became progressively less pronounced. The automated platelet count became normal within 30 days.

**FIGURE 1:**
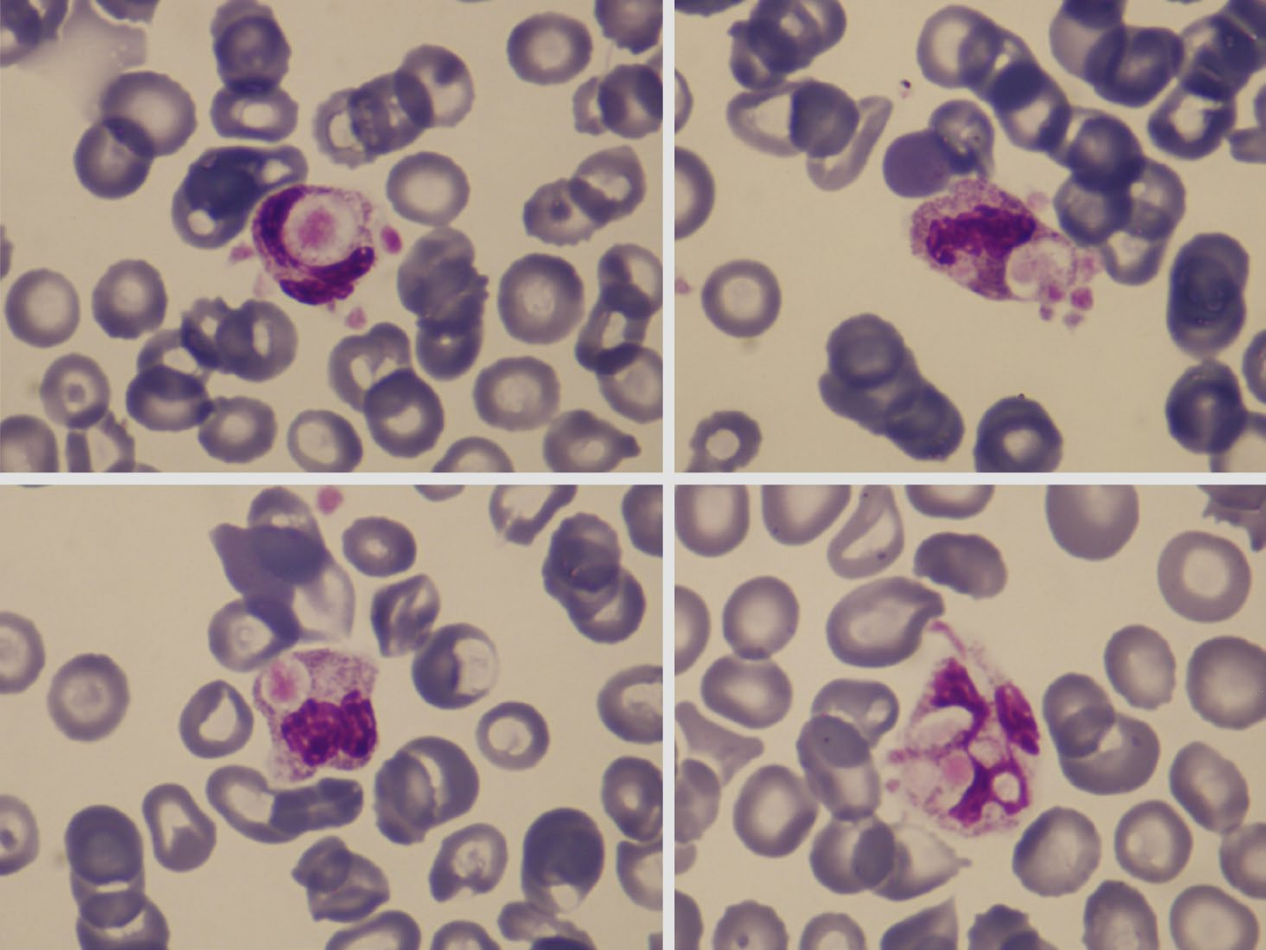
Four fields of the peripheral‐blood smear, showing ingestion of platelets by neutrophilic granulocytes (May‐Grünwald‐Giemsa stain, ×1000).

A peripheral‐blood smear should always be examined in new cases of thrombocytopenia or whenever the platelet count is unexpectedly low, in order to confirm the thrombocytopenia. The blood smear may be requested by physicians or initiated by laboratory staff [1]. As seen here, a laboratory‐initiated blood smear, is particularly valuable because it may permit earlier recognition of pseudothrombocytopenia. The incidence of pseudothrombocytopenia is 1.9% among hospitalized patients and 0.15% in the outpatient setting [2]. Falsely low counts may be the result of small clots, platelet clumping, platelet satellitism, or abnormally large platelets. Phagocytosis of platelets by neutrophilic granulocytes is a rare cause of pseudothrombocytopenia, often seen in association with platelet satellitism [2]. Ordinarily, “flags” produced by automated blood‐count analyzers are very useful for detecting errors in platelet enumeration. In this patient, however, the flagging system did not detect an error, suggesting that phagocytosis of platelets by neutrophils cannot be detected by the automated flagging systems.

Phagocytosis of platelets by neutrophilic granulocytes is an in vitro phenomenon occurring only in EDTA‐anticoagulated blood [2, 3]. It is not reproduced if blood is drawn into a citrated tube or smears are made directly from capillary blood. The underlying mechanism is not fully understood, but might be related to IgG autoantibodies directed against the glycoprotein IIb/IIIa complex of platelets and the Fcγ‐receptor III of neutrophils. The working hypothesis is that at room temperature, the chelation of calcium ions by EDTA alters the glycoprotein IIb/IIIa molecule and the neutrophil Fcγ‐receptor exposing epitopes for the IgG autoantibody, which forms a bridge between platelets and neutrophils. Neutrophil–platelet adherence is followed by platelet phagocytosis [2, 3]. Platelet activation may also play a part. On activation by inflammatory triggers, platelets express P‐selectin on their surfaces, which facilitates platelet adherence to neutrophils [4].

In contrast to platelet clumping which frequently occurs in routine blood counts, platelet phagocytosis is seen mainly during severe illness such as infection, thrombosis, and malignant hypertension [3]. Our patient's case is, to our knowledge, the first report of pseudothrombocytopenia occurring in pseudomembranous colitis.

This case illustrates an important cause of spurious thrombocytopenia in the acutely ill patient. Awareness of this phenomenon can prevent unnecessary measures such as platelet transfusions, postponement of invasive interventions, or discontinuation of medications.

## Author Contributions

All authors were involved in the care of the patient and have contributed to the writing of the manuscript. All authors agree to the submission of this manuscript to the *American Journal of Hematology*.

## Consent

The patient has provided consent.

## Conflicts of Interest

The authors declare no conflicts of interest.

## Data Availability

The original data of this study can be obtained via email to Konstantinos Liapis (kosliapis@hotmail.com).
